# Targeting the Mammalian Target of Rapamycin (mTOR) in Cancer Therapy: Lessons from Past and Future Perspectives

**DOI:** 10.3390/cancers3022478

**Published:** 2011-05-24

**Authors:** Marc Dufour, Anne Dormond-Meuwly, Nicolas Demartines, Olivier Dormond

**Affiliations:** Department of Visceral Surgery, Centre Hospitalier Universitaire Vaudois and University of Lausanne, Pavillon 3, Av. de Beaumont, Lausanne 1011, Switzerland; E-Mails: marc.dufour@chuv.ch (M.D.); anne.meuwly-dormond@chuv.ch (A.D.-M.); demartines@chuv.ch (N.D.)

**Keywords:** cancer, mTOR, rapalogs, signaling, therapy

## Abstract

Over the last decade, extensive studies have been made to understand the role played by the mammalian target of rapamycin (mTOR) in cancer. Knowledge in this field has been gained from discoveries in basic research as well as from observations made in patients treated with allosteric mTOR inhibitors such as rapamycin. Despite promising preclinical studies, targeting mTOR in cancer therapy has shown limited clinical benefits so far. However, recent findings have revealed the complexity of the functions of mTOR in cancer and have helped develop new strategies to improve the anticancer efficacy of mTOR inhibitors. In particular, a complex network between mTOR and other signaling pathways has been identified that influences the anticancer efficacy of mTOR inhibitors. In addition, an emerging role of mTOR in the tumor microenvironment has been suggested. In this review, we confront the major findings that have been made in the past, both in experimental settings as well as in clinical trials. We further review the strategies that have been designed to further improve the efficacy of therapies targeting mTOR.

## 1. Introduction

Tumor development results from complex processes that enable a tumor to grow and metastasize. Several hallmarks have been proposed as necessary for tumor development. They include: Sustaining proliferative signaling, evading growth suppressors, resisting cell death, enabling replicative immortality, inducing angiogenesis, activating invasion and metastasis, reprogramming energy metabolism and avoiding immune destruction [1]. Interestingly, the mammalian target of rapamycin (mTOR) has been shown to play a role in several aspects of these hallmarks and, accordingly, represents an important player in the biology of tumors. Indeed, mTOR regulates cell growth, proliferation and survival and is at the crossroads of different signaling pathways that are frequently mutated in various types of cancer [2]. As a consequence, the resulting over-activation of mTOR leads to a sustaining of proliferative signals and resistance to cell death. Furthermore, a function for mTOR in cancer invasion and metastasis has also been demonstrated and mTOR plays an emerging role in cell metabolism [3]. In addition to the proliferating cancer cells, tumors are also composed of a microenvironment that contains distinct cell types, including endothelial cells, innate and adaptive immune cells and fibroblasts that influence the growth of a tumor. The role of mTOR in these cells has also been partially characterized. In fact, mTOR is an important mediator of tumor angiogenesis and the immunosuppressive effects of mTOR inhibitors has been known for decades and is being exploited in transplanted patients [4,5]. Hence, mTOR influences tumor growth by playing a global role that is not restricted to cancer cell proliferation and survival but that also affects the angiogenic and the immunological responses found in the microenvironment of a tumor.

A first round of clinical trials in cancer patients has been performed, evaluating the anticancer efficacy of rapamycin (sirolimus) and rapamycin like drugs (rapalogs) including RAD001 (everolimus), CCI-779 (temsirolimus) and AP23573 (deferolimus). Overall, rapalogs were less successful than expected, generating objective responses in few cancers. Although these clinical trials may challenge the relevance of targeting mTOR in cancer therapy, recent findings have demonstrated the complex consequences of blocking mTOR in cancer cells and have helped develop new therapeutic strategies aimed to improve the anticancer efficacy of mTOR inhibitors. Here, we review the role played by mTOR in cancer biology. We further analyze the knowledge that we have obtained from the use of mTOR inhibitors in clinical and in basic research. Finally, we enumerate new strategies of mTOR-based therapies in oncology.

## 2. mTOR Signaling Pathway in Cancer

**Figure 1.**
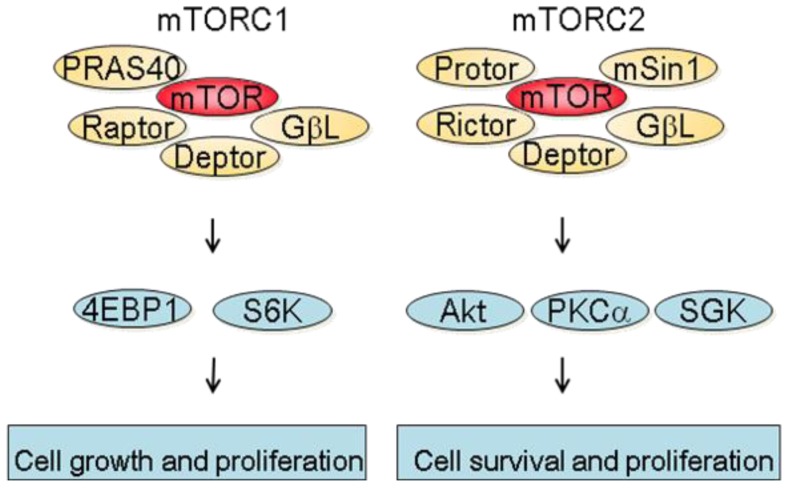
mTOR (the mammalian target of rapamycin ) and its two complexes mTORC1 and mTORC2. mTOR is part of two functionally distinct complex; mTORC1 and mTORC2. mTORC1 is composed of mTOR, PRAS40, Raptor, Deptor and GβL and regulates among others cell growth and proliferation by phosphorylating 4E-BP1 and S6K. mTORC2 is composed of Protor, Rictor, Deptor, GβL and mSin1 and regulates cell survival and proliferation by activating Akt, PKC α and SGK.

mTOR is a 289 kDa protein that belongs to the phosphatidylinositol 3-kinase-related kinase family [6]. mTOR exerts its biological functions as being part of two different protein complexes; the mTOR complex 1 (mTORC1) and the mTOR complex 2 (mTORC2). mTORC1 is composed of Raptor, mTOR, mLST8, Deptor and PRAS40 and is regulated by oxygen levels, amino acids, energy and growth factors. Upon activation, mTORC1 participates in translation initiation and protein synthesis by phosphorylating at least two well-characterized effectors, S6K1 and 4E-BP1 [3]. In addition, one major consequence of mTORC1 inhibition is the downregulation of several mRNA coding for proteins implicated in the G1-S phase progression. This results in reduced cell proliferation by inducing a G1 arrest and accounts in part for the anti-proliferative properties of mTOR inhibitors [7]. mTORC2 consists of Rictor, mTOR, mLST8, Protor, Deptor as well as mSin1 and is regulated by growth factors. Downstream effectors of mTORC2 are Akt, which regulates cell proliferation and survival, and PKC alpha, which controls cytoskeletal organization [8]. mTORC2 also activates SGK1, however the functional significance of this activation needs to be further characterized (Figure 1).

**Figure 2.**
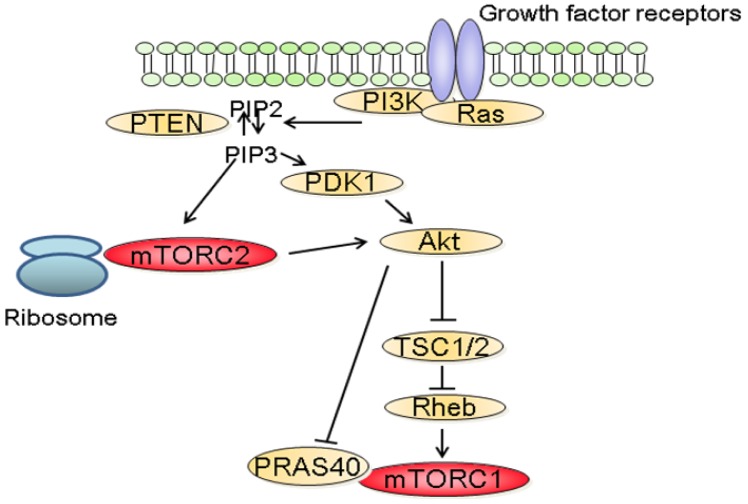
Growth factors mediated mTORC1 and mTORC2 activation. The stimulation of growth factor receptors by their ligands leads to the activation of PI3K wich catalyzes the formation of phosphoinositide-3,4,5-tri-phosphate (PIP3). This triggers the translocation of Akt to the membrane where it gets activated by the phosphoinositide-dependent kinase 1 (PDK1). In addition, the activation of Akt requires the phosphorylation by mTORC2. Once activated Akt positively regulates mTORC1 by either inhibiting PRAS40 or TSC2, two negative regulators of mTORC1. mTORC2 activation requires its association to the ribosomes which is enhanced by PI3K activity. Finally, the lipid phosphatase PTEN acts as a negative regulator of this pathway by converting PIP3 into PIP2.

The molecular mechanisms that regulate the activation of mTORC1 have been extensively studied. Among these, the phosphatidylinositol-3 kinase (PI3K)/Akt signaling pathway has been identified as a major mediator of growth factors-induced mTORC1 activation (Figure 2) [3,6]. Following stimulation with growth factors, PI3K is activated and catalyzes the formation of phosphoinositide-3,4,5-tri-phosphate (PIP3) resulting in the recruitment to the plasma membrane and to the activation of Akt. In turn, Akt inactivates TSC2, a large protein that is part of the TSC1-TSC2 complex. Inactivation of the TSC1/2 complex leads to the activation of the small GTPase Rheb which stimulates the kinase activity of mTORC1 [3]. In addition to its effect on TSC2, Akt also inactivates PRAS40 preventing it from blocking mTORC1 activity [9]. In parallel to the PI3K/Akt axis, growth factors also stimulate mTORC1 activity through the Mek/Erk signaling pathway [10]. Phosphorylation of TSC2 by Erk or phosphorylation of raptor by p90RSK, a downstream effector of Erk, have both been proposed to explain the activation of mTORC1 by Erk signaling pathway [11].

The molecular mechanisms by which mTORC1 is regulated by stimuli other than growth factors have also been partially described [3]. Low energy levels result in decreased AMP/ATP ratio which leads to the activation of AMPK. AMPK phosphorylates and activates TSC2 inducing the inhibition of mTORC1 [12]. Furthermore, it has also been suggested that AMPK decreases the kinase activity by directly phosphorylating raptor, inducing its binding to 14-3-3. Of clinical importance, LKB1 a protein mutated in the Peutz-Jeghers syndrome, is an upstream activator of AMPK [13]. Hypoxia also inhibits mTORC1 activity either by reducing ATP levels and thus activating AMPK or by inducing the expression of REDD1 that inhibits mTORC1 by stabilizing the TSC1/TSC2 complex [14]. Amino acids signaling to mTORC1 involves the recruitment of mTORC1 to lysosomal membranes. In turn, mTORC1 associates with Rag GTPases which promotes its interaction with the lysosomal pool of Rheb [15,16]. Finally, DNA damage also downregulates mTORC1 activity in a p53-dependent process which involves transactivation of negative regulators of mTORC1, such as TSC2 and AMPK [17].

In contrast to mTORC1, little is known about the upstream regulators of mTORC2. Recent findings suggest that the activation of mTORC2 requires its association with ribosomes and that this association requires PI3K activity (Figure 2) [18]. In addition, PIP3, the product of PI3K also directly stimulates the activity of mTORC2 in kinase assay [19]. The role of the ribosome has however not been characterized in this context.

Dysregulation of the PI3K/Akt/mTOR signaling pathway is common in human cancers [2,7]. Gain of function mutations of the catalytic as well as the regulatory subunits of PI3K have been identified in a variety of tumors [20,21]. Similarly, activating mutations of AKT have been described in cancers [22,23]. In addition, since PI3K signals are often activated by receptor tyrosine kinases (RTK), aberrant PI3K signaling is also observed in cancers harboring mutations of RTK. Furthermore, as PI3K is also a downstream effector of the small GTPase Ras, increased PI3K activity is also common in cancer with Ras mutations [24]. Finally, loss of function of the tumor suppressor phosphatase and tensin homolog deleted on chromosome 10 (PTEN) is frequently reported in cancers [25]. As physiologically, PTEN terminates PI3K signals by dephosphorylating PIP3 into phosphatidylinositol 4,5-bisphosphate (PIP2), the inactivation of PTEN observed in tumors results in the activation of PI3K/Akt signaling.

Since mTOR plays a central role in cell growth and proliferation and since it is frequently over activated in tumor cells, mTOR represents an ideal target in cancer therapy. Indeed, several clinical trials have already been performed and have evaluated the anticancer potential of mTOR inhibitors.

## 3. mTOR and Cancer; What We Have Learned From Clinical Trials

The effect of mTOR inhibition in clinical trials has been extensively studied using rapalogs that specifically inhibit mTORC1. Rapalogs interact with the immunophilin FK506-binding protein (FKBP12) and together bind the FKBP12-rapamycin binding domain of mTOR, resulting in the inhibition of mTORC1 [26]. Notably, recent studies have shown that only certain functions of mTORC1 are targeted by rapalogs [27]. In contrast to mTORC1, mTORC2 is insensitive to short-term exposure of rapalogs. However, depending on the cell type, prolonged exposure to rapalogs also inhibits mTORC2 [28].

Preclinical studies have shown that rapalogs exert their anti-cancer effects in part by inhibiting cell proliferation. Rapalogs reduce the synthesis of proteins involved in cell cycle progression resulting in a G_1_ cell cycle arrest [7]. At the molecular level, inhibition of mTORC1 induces the dephosphorylation of 4E-BP1which in turn binds eIF4E, preventing it from interacting with the cap structure of the 5′ untranslated regions of mRNA. Consequently, cap-dependent translation initiation is impaired, particularly affecting mRNAs with highly structured 5′untranslated regions, like those encoding for cyclin D1, c-myc or VEGF [29]. Thus, 4E-BP1 appears to be an important target of mTOR that influences cancer growth. Indeed, rhabdomyosarcoma cells harboring very low level of 4E-BP1 are resistant to the growth inhibitory effects of rapamycin [30]. In addition to their effects on 4E-BP1, rapalogs also inhibit the G_1_-S cell cycle progression by blocking the phosphorylation of the retinoblastoma protein (pRb). On one hand, rapalogs decrease the expression of cyclin D1, therefore reducing the level of active cyclin-dependent kinase (cdk)/cyclin D1 complexes which regulate pRb phosphorylation. On the other hand, rapalogs also up-regulate p27 expression which negatively affects cdk [31,32].

Following encouraging pre-clinical studies, the anticancer efficacy of rapalogs has been less successful than expected in the clinic [33]. To date, rapalogs have been approved for the treatment of advanced renal cell carcinoma. Patients with poor-prognosis renal cell carcinoma receiving temsirolimus had a significantly longer overall survival and progression-free survival compared to patients treated with interferon [34]. Similarly, in renal cell carcinoma patients who failed prior therapy, everolimus significantly extended the progression free survival [35]. In addition to advanced renal cell carcinoma, rapalogs showed also efficacy in mantle cell lymphoma. Temsirolimus significantly increased progression-free survival compared to the investigator's choice therapy in patients with refractory mantle cell lymphoma [36]. Finally, more recently, everolimus also prolonged progression-free survival among patients with progressive advanced pancreatic neuroendocrine tumors [37]. Interestingly, the use of mTORC1 inhibitors in these types of cancer relies also on a biological rationale. Indeed, renal cell carcinoma is a highly vascularized tumor due to the excessive production of vascular endothelial growth factor (VEGF). At the molecular level, the production of VEGF is a consequence of the accumulation of the hypoxia-inducible factor alpha (HIF-α). Since mTORC1 regulates HIF-α expression, the antitumor effects of rapalogs in renal cell carcinoma appear to be due to the inhibition of vessel formation in these tumors [38]. Similarly, the genetic hallmark of mantle cell lymphoma is the chromosomal translocation t(11;14) resulting in the accumulation of cyclin D1 and cell cycle progression [39]. As translation of cyclin D1 mRNA depends on mTORC1 activity, mTORC1 inhibition reduces cyclin D1 accumulation. Finally, PTEN is frequently down regulated in pancreatic endocrine tumors resulting in the activation of the PI3K/Akt/mTOR pathway [40].

In addition, rapalogs are being evaluated in phase I-III clinical trials for several other cancers including ovarian, endometrial, glioblastoma, bladder, prostate cancer as well as hematological malignancies [41]. Given the high prevalence of PI3K/Akt/mTOR activation resulting from loss of PTEN expression, a strong rationale exists to use mTOR inhibitors in prostate cancer [42,43]. Several clinical trials are ongoing but preliminary analysis have shown that rapamycin is well tolerated in prostate cancer patients and decreases the level of mTORC1 activity in the tumor [44,45]. However, there was no change in Ki-67 or cleaved caspase-3 staining, (respectively markers of cell proliferation and cell death) in prostate tumor cells of patients treated with rapamycin for 14 days [45]. In addition, in another pilot study, rapamycin also achieved limited clinical responses in patients with hormone-refractory prostate cancer [46]. Responses were observed in 2 to 12 patients and stable disease was noted in 4 of 12 patients. These results suggest that although mTOR represents a promising target in prostate cancer patients, future studies are needed to define which patients will likely respond to rapamycin.

mTOR signaling pathway is also deregulated in several hematological malignancies including acute and chronic myeloid leukemia and multiple myeloma [47,48]. Following encouraging pre-clinical results, rapalogs were tested in patients with relapsed/refractory acute myeloid leukemia. In a small study, nine patients received rapamycin for 28 days. A 50% reduction of blood or bone marrow blasts was reported in 4 patients [49]. Partial or total responses were also reported in 22% of patients receiving rapamycin in combination with an etoposide-based chemotherapy [50]. In chronic myeloid leukemia, the fusion protein BCR-ABL is the trigger of the disease and is specifically targeted by tyrosine kinase inhibitors such as imatinib. Of note, BCR-ABL-mediated mTOR activation is involved in the progression of the disease and studies have shown that blocking mTOR might be promising in patients that are resistant to imatinib [51]. In multiple myeloma, emerging evidence demonstrates that mTOR is an important mediator of cell proliferation and survival [52]. In fact, rapalogs are being evaluated clinically and a phase II trial has reported an overall response rate of 38% in patients with relapsed multiple myeloma [53]. Finally, the effects of rapalogs are also being investigated in acute lymphoid leukemia and in chronic lymphocytic leukemia. In particular, in chronic lymphocytic leukemia, clinical responses were observed in 4 out of 22 patients [54] and clinical responses were also reported in another phase II trial that was stopped due to toxicities of rapamycin [55].

Finally, therapies that indirectly inhibit mTOR have also shown clinical promises in cancer therapy and further underline the importance of mTOR in cancer biology. For example, the biguanide metformin used to treat type 2 diabetes has emerged as an anticancer agent. Indeed, several retrospective epidemiological studies have demonstrated a reduced cancer risk in diabetic patients treated with metformin [56-58]. In addition, prospective clinical trials have also been initiated in non-diabetic patients to evaluate the anticancer efficacy of metformin. Preliminary analysis have shown that metformin possesses favorable effects on tumor cell proliferation and apoptosis [59]. Several mechanisms have been proposed to explain the anticancer activity of metformin. Among those, metformin directly reduces the growth of cancer cells *in vitro* [56]. At the molecular level, metformin induces the activation of AMPK which results in the inactivation of mTORC1. In addition, metformin also increases the expression of REDD1 which inhibits mTORC1 [60]. Thus, these studies suggest that the anticancer efficacy of metformin relies in part on its ability to inhibit mTORC1 [61]. Future studies will further explore the effects of metformin in cancer patients and further elucidate the role of metformin induced-mTORC1 inhibition in these effects.

In summary, despite numerous clinical trials, few cancers have responded to rapalogs. Furthermore, the benefits of rapalogs were limited in these cancers. These studies also underline the importance of identifying biomarkers capable of predicting patients that are likely to respond to rapalogs. Although preclinical studies have suggested that mutations in the PI3K/Akt pathway, such as loss of PTEN expression, render tumors more sensitive to rapalogs, no reliable biomarker has been identified in patients [62]. The use of new technologies such as gene expression or phosphoproteomic profiling will probably help identify new biomarkers [63]. Such clinical analysis using high-throughput genomics has already been initiated in the context of renal cell carcinoma treated with everolimus [64]. Finally, in addition to predictive biomarker, pharmacodynamic biomarkers are also needed to assess the efficacy of rapalogs as well as to identify the active doses. Phosphorylation of downstream effectors of mTORC1 has been proposed as pharmacodynamic markers and will need to be confirmed in larger studies [65].

## 4. mTOR and Cancer; What We Have Learned from the Use of Rapamycin in Transplant Patients

The development of cancer is a major concern in transplant patients following immunosuppression with an overall risk of cancer increased by three to fivefold [66]. Skin cancers, Kaposi's sarcoma and lymphoproliferative disease are the most predominant post transplant malignancies. Transplant recipients are at increased risk of cancer as immunosuppression impairs immune response against tumor cells. In addition, some of the immunosupressive drugs also promote tumor growth by increasing angiogenesis and cancer cell aggressiveness [67,68]. Therefore, in this context, a therapeutical approach to prevent graft rejections and tumor development is to use rapalogs that have both immunosuppressive and anticancer effects [69]. Very few prospective studies have analyzed the effect of rapalogs on cancer development in transplanted patients as a primary endpoint. Nevertheless, a single center prospective randomized trial has shown that, in renal transplant recipients that had developed premalignant skin lesions, conversion of their immunosuppression to rapamycin stopped the progression of the lesion or induced its regression. In addition, rapamycin also reduced the incidence of nonmelanoma skin cancer in these patients [70]. A lower incidence of malignancy was also reported in a larger cohort of kidney recipients following the conversion of the immunosuppression to rapamycin [71]. Finally, case studies have also shown that rapamycin reduce the incidence of cancers in transplanted patients [72]. In addition, the efficacy of rapamycin was also reported on the regression of established tumors. Indeed, several case studies have reported that rapamycin is effective in treating Kaposi's sarcoma in transplanted patients [73,74]. As Kaposi's sarcoma is a highly vascularized tumor, rapamycin might be particularly efficient due to its anti-angiogenic property [4,75]. Interestingly, other mechanisms have also been proposed to explain the efficacy of rapamycin in these cancers such as reduced immunosuppression following conversion to rapamycin. However, more importantly, it was reported that, in two liver recipients that had developed Kaposi's sarcoma, conversion to rapamycin was associated with the recovery of CD4 and CD8 memory T cells against human herpes virus 8 that is responsible for the disease [76]. Furthermore, no recovery of CD4 memory T cells was observed in a kidney recipient for whom rapamycin failed to induce tumor regression. Transplanted patients treated with rapamycin had also better outcome with regard to cytomegalovirus infection compared to patients with standard immunosuppression, suggesting that rapamycin might enhance the immune response [77]. These observations confirm recent findings that suggest that rapamycin have dichotomous effects in immunobiology. Indeed, emerging data support immunostimulatory properties of rapalogs. Rapamycin induces immunostimulatory effects on CD8^+^ memory T cell response after pathogen infection [78,79] and also promotes CD8^+^ T cell-mediated antitumor activity [80]. In contrast, rapalogs have been used for a long time as immunosuppressive agents as they inhibit the proliferation of T cell following antigen stimulation [81]. In addition, rapamycin also increases the generation of T regulatory cells, a subset of T cells that promotes tolerance to an antigen [82]. Furthermore, the immunodepressive functions of mTORC1 inhibitors were also supported by their inhibitory effects on dendritic cells [83]. Therefore, it appears that rapalogs can exert both immunosuppressive and immunostimulatory effects. Future studies will help clarify the conditions and parameters regulating these aspects of mTORC1 inhibitors. Clearly, avoiding the immunosuppressive while increasing the immunostimulatory properties of mTORC1 inhibitors will influence the anticancer efficacy of mTORC1 inhibitors.

Finally, patients treated with rapalogs are also at an increased risk of developing interstitial pneumonitis and other inflammatory-related complications [84]. Histologically, this pneumonitis is characterized by neutrophils and CD4 T helper cells infiltrates in the absence of infection. Consistent with this observation, proinflammatory properties of mTORC1 inhibitors have also been described in various experimental models [85]. Emerging data has shown that the PI3K/Akt/mTOR pathway plays an important role in regulating inflammatory mediators in myeloid cells by limiting the inflammatory responses in dendritic cells and macrophages [85]. Moreover, mTOR reduces tissue infiltration by leukocytes by diminishing the expression of the adhesion molecule ICAM-1 by the endothelium [86]. Taken together, these observations show that mTOR inhibition promotes inflammation. As inflammatory cells play a pivotal role in the tumor microenvironment, future studies will define the effect of mTOR inhibitors on inflammatory cells present in the tumor microenvironment.

## 5. mTOR and Cancer; What We Have Learned from Basic Research

**Figure 3.**
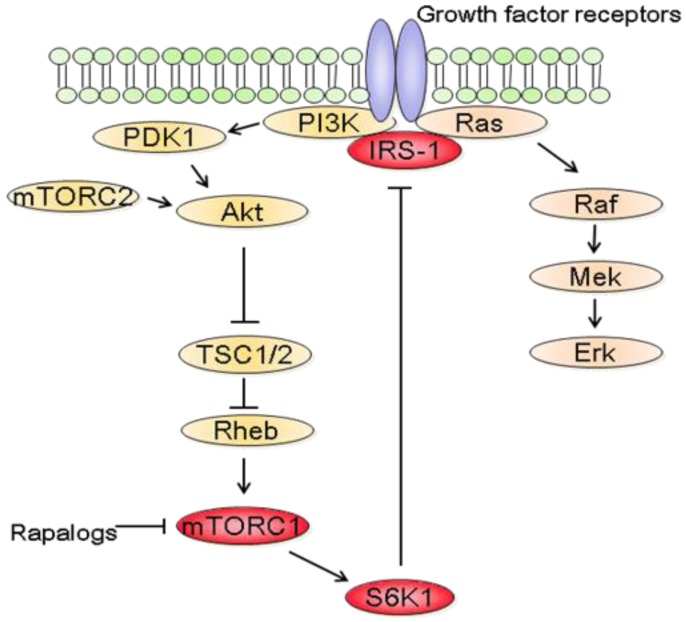
mTORC1 inhibition activates proliferative and prosurvival pathways. Activation of mTORC1 leads to the inhibition of PI3K through a negative feedback loop that involves S6K1. The negative feedback loop is blocked following mTORC1 inhibition by rapalogs. This leads to the activation of PI3K/Akt and Raf/Mek/Erk proliferative and prosurvival signals that counteract the anticancer efficacy of rapalogs.

In parallel to the clinical trials, progresses have been made in mTOR biology. In addition to the identification of two distinct complexes, mTORC1 and mTORC2, experimental studies have also revealed a complex network between mTOR and other signaling pathways [87]. It thus became clear that the inhibition of mTORC1 induces the activation of other prosurvival signals that counteract their anticancer efficacy (Figure 3). In fact, rapalogs block a negative feedback loop whereby activation of mTORC1 and its downstream effector S6K1 reduces PI3K/Akt activity [87]. At the molecular level, down regulation of insulin receptor substrate 1 (IRS-1) by S6K1 as well as down regulation of platelet-derived growth factor have been involved in this negative feedback loop [88,89].

Interestingly, rapalogs-mediated Akt activation has also been described in patients treated with the rapalog RAD001. Biopsies of liver metastases or skin lesions of patients with colon or breast carcinoma were analyzed before and after 4 weeks of treatment with RAD001. Immunhistochemistry studies revealed that the levels of Akt phosphorylation were higher following RAD001 treatment [90]. Similarly, Akt phosphorylation was analyzed in various tumors of patients receiving RAD001 [91]. Akt phosphorylation was increased in 50% of the treated tumors. Increased Akt phosphorylation was also noted in skin biopsies [91]. The effect of rapamycin on Akt phosphorylation was also assessed in patients with recurrent glioblastoma which lacked PTEN expression [92]. Rapamycin led to increased Akt phosphorylation in tumors in seven of fourteen patients. Akt activation was also associated with shorter time-to-progression in these patients suggesting that combining rapalogs with inhibitors that block Akt activity may be useful in patients. This study also illustrates the difficulties of predicting the effect of rapalogs on Akt as only 50% of the patients had increased Akt phosphorylation in the tumor following rapamycin treatment although all patients suffered from glioblastoma.

In addition to the feedback loop connecting mTORC1/S6K1 to PI3K/Akt, recent studies have also shown that blocking mTORC1/S6K1 with rapalogs activates the Raf/Mek/Erk signaling pathway in a PI3K dependent manner [93]. Treatment of cancer cell lines with rapamycin increased Mek/Erk activity and pharmacological blockade of Mek increased the growth inhibitory efficacy of rapamycin [93,94]. Most importantly, rapalogs-mediated Erk activation was also reported in patients. Immunohistochemical analysis of tumor biopsies before and after RAD001 treatment showed that Erk phosphorylation was increased following treatment [93]. It is also worth noting that rapalogs induced Erk activation is cell type dependent. For example, treatment of HCT-116, MCF-7 or DU145 cells with rapamycin did not increase Erk phosphorylation [94]. Furthermore, combination therapy using rapamycin and Mek inhibitors decreased the growth of prostate cancer cells more effectively than either agent alone, although rapamycin did not increase Erk phosphorylation in these cells [95]. This suggests that Mek inhibitors would potentiate the anticancer efficacy of rapalogs regardless of whether rapalogs increase Erk activity. Therefore, it might be more relevant in identifying tumors that will respond to combined mTOR and Mek inhibitions rather than determining the tumors for which mTORC1 inhibition leads to Erk activation. Of relevance to cancer therapy, mTOR inhibition also increases Mek/Erk signaling in endothelial cells [96]. Targeting mTOR and Mek simultaneously has additive anti-angiogenic effects both *in vitro* and *in vivo*. Therefore combining mTOR and Mek inhibitors might have synergistic effects both on tumor cells and on the tumor vasculature.

In addition to the activation of PI3K/Akt and Mek/Erk signaling pathways, rapalogs induce other signals that might compromise the antitumor effects of rapalogs. For example, mTORC1 inhibition leads to the paradoxical phosphorylation of eIF4E in several types of cancer cells [97]. eIF4E regulates the initiation translation of mRNA with 5′ untranlsated regions including a number of transformation related genes and has been identified as a potential oncogene [98]. Notably, rapalogs mediated eIF4E phosphorylation was dependent on Mnk (MAP kinase interacting kinase) and the inhibition of Mnk increased rapamycin-mediated growth inhibition [97]. Finally, it has also been reported that rapamycin-activated JNK signaling pathway in colon cancer cells plus combined JNK and mTOR inhibition had additive anti-tumor effects [99]. Future studies will explore the relevance of rapalogs mediated eIF4E or JNK activation in a clinical setting.

## 6. Enhancing the Anticancer Efficacy of mTORC1 Inhibitors

As mentioned earlier, rapalogs used as single agents have shown very limited benefits in cancer therapy. This might be partly explained by the observation that rapalogs induce the activation of other signaling pathways, resulting in proliferative and prosurvival signals that impede the anticancer efficacy of rapalogs. Therefore, combining rapalogs with other agents implicated in these feedback activations should improve the efficacy of rapalogs in cancer therapy.

In this context, preventing rapalogs-mediated PI3K/Akt activation has already shown promising effects. Since growth factor receptors act upstream of the PI3K/Akt signaling pathway and have been involved in mTORC1-mediated PI3K/Akt activation, combining growth factor receptor inhibitors with rapalogs is a reasonable strategy. Indeed combining IGF-1R inhibitors with rapalogs exert additive antiproliferative effects compare to single treatment in prostate, breast cancer and myeloma cells [90,100]. Interestingly, this strategy is already under clinical evaluation [101]. Targeting other growth factor receptors such as EGFR with rapalogs also results in enhanced anticancer efficacy [102,103].

Another strategy to increase the anticancer efficacy of rapalogs is to combine rapalogs with PI3K inhibitors. In fact, PI3K appears to be a central player in the feedback activation mediated by rapalogs. As mentioned earlier, mTORC1 inhibition-mediated Akt, Erk and Mnk activation is PI3K dependent. Such a strategy has already been tested in experimental models and has shown that LY294002, a PI3K inhibitor, enhanced the growth inhibitory effects of rapalogs in lung cancer cells as well as in T-cell leukemia cells [94,104]. While LY294002 cannot be used in the clinic due to its high toxicity, several other PI3K inhibitors have been developed and are under clinical evaluation [105].

Akt inhibitors have also been developed and tested in association with rapalogs. It was reported that the Akt inhibitor perifosine, in combination with nanoparticle bound rapamycin had increased anti-tumoral activity compared to either agent alone in multiple myeloma [106]. Furthermore, targeting downstream effectors of Akt such as Foxo proteins may also potentiate the anticancer efficacy of rapalogs [107].

As mentioned earlier, mTORC1 inhibition leads to the activation of Erk and targeting both Erk and mTOR signaling pathways has additional antitumoral and anti-angiogenic effects [96,108]. Consistent with these findings, combining rapalogs with sorafenib, which blocks Raf an upstream activator of Erk, exerted an enhanced anti-tumoral effect compared to rapalogs or sorafenib alone [109,110].

Finally, although the above mentioned strategies to improve rapalogs anticancer efficacy rely on a biological rationale, it is worth noting that rapalogs efficacy can be enhanced by molecules that have no obvious link with mTOR signaling pathway. For example, the anticancer activity of rapalogs is improved by vorinostat a histone deacetylase inhibitor [111]. Furthermore, DNA methyltransferase inhibitors also potentiate the anticancer efficacy of rapamycin in colon cancer cells [112].

In summary, several new therapeutic strategies have improved the anticancer efficacy of rapalogs in experimental models. Ongoing clinical trials will reveal their efficacy and tolerability in patients.

## 7. Enhancing mTOR Targeted Therapies; ATP-Competitive Inhibitors of mTOR

The identification that mTOR acts in two distinct complexes, mTORC1 and mTORC2, and that mTORC2 is insensitive to rapamycin has led to the rapid development of inhibitors able to block both mTORC1 and mTORC2 [113,114]. It was speculated that blocking both complexes would produce stronger anticancer effects than rapalogs. Indeed, mTORC2 exerts an emerging role in cancer development and has been shown to participate in prostate and colon cancer progression [26,115]. Several such inhibitors have been developed and characterized [114]. Acting as ATP-competitive inhibitors, they either specifically block mTORC1 and mTORC2 or induce a dual inhibition PI3K/mTOR. The anticancer efficacy of these inhibitors has been tested in various experimental tumor models [38,116]. Overall, the anticancer and anti-angiogenic efficacies of ATP-competitive inhibitors of mTOR are superior to those of rapamycin [116].

Indeed, AZD8055, a specific mTOR inhibitor, showed antiproliferative effects in various cancer models that were superior to those of rapamycin. Interestingly, cancer cells that did not respond to rapamycin were sensitive to AZD8055 [117,118]. Similarly, WYE-132 that blocks both mTORC1 and mTORC2 induced a stronger inhibition of cancer cell growth and survival compared to temsirolimus [119]. Of note WYE-132 but not temsirolimus also achieved regression of tumors *in vivo* through the induction of tumor cell apoptosis, suggesting that mTOR kinase inhibitors might have cytolytic properties [119]. Furthermore, targeting mTORC1 and mTORC2 with the chemical compounds OSI-027 or OXA-01 reduces tumor growth more efficiently than rapamycin [120]. The anti-angiogenic effects of OSI-027 and OXA-01 were also superior to those of rapamycin. Finally, the inhibition of mTORC1 and mTORC2 with PP242 in leukemic cells that express the BCR-ABL oncogene, results in a strong anti-leukemic activity that is superior to rapamycin [121].

As mentioned earlier, dual mTOR/PI3K inhibitors have also been developed. These drugs target the ATP binding sites of both mTOR and PI3K and therefore block mTORC1, mTORC2 as well as PI3K. Several dual inhibitors such as NVP-BEZ235 have already been tested and have shown antitumor activity in numerous preclinical cancer models [116]. Interestingly, NVP-BEZ235 displayed anti-tumor efficacy in cancer models harboring PI3K mutations but not K-Ras mutations underlining the importance to define biological markers that will predict the response to these drugs [122,123]. In addition, the anti-cancer efficacy of NVP-BEZ235 has also been compared to rapamycin. In renal cell carcinoma, NVP-BEZ235 reduced the growth of tumor xenografts more efficiently than rapamycin [124]. NVP-BEZ235 also induced tumor cell apoptosis which was not observed with rapamycin. NVP-BEZ235 has been tested in pre-clinical models of hematological malignancies. It significantly reduces acute myeloid leukemia cell proliferation and survival [125]. In addition, NVP-BEZ235 also showed anti-tumoral activity in Waldenstrom macroglobulinemia and T-cell acute lymphoblastic leukemia [126,127]. Other dual PI3K/mTOR inhibitors have been evaluated and consistently showed the inhibition of tumor growth [116].

Of note, ATP-competitive inhibitors of mTOR have also shown efficacy in tumor models of genetically engineered mice, which may reflect the pathogenesis of human cancers more closely than xenograft models. In a mouse model of lung adenocarcinomas generated by the expression of a constitutively active mutant of the catalytic subunit of PI3K, a marked tumor regression was observed following treatment with NVP-BEZ235 [128]. Similarly, in a transgenic murine ovarian cancer model triggered by the expression of an active K-ras mutant and the loss of PTEN expression, mice treated with NVP-BEZ235 survived significantly longer [129]. Finally, AZD8055, a specific mTORC1/mTORC2 inhibitor reduced the growth of B-cell follicular lymphoma that develops in PTEN-deficient mice [130].

At the molecular level, several mechanisms explain the greater anitumoral efficacy of ATP kinase inhibitors of mTOR compared to rapalogs. ATP-competitive inhibitors not only inhibit mTORC2 but have also a broader inhibitory effect on mTORC1. Indeed, the proliferation of mouse embryo fibroblasts is slightly reduced by rapamycin while it is totally blocked by Torin 1 or PP242, two ATP-competitive inhibitors of mTOR [131,132]. A similar response is observed in mouse embryo fibroblasts lacking mTORC2 activity, showing that the stronger efficacy of mTOR kinase inhibitors was a consequence of a more pronounced inhibition of mTORC1 rather than the inhibition of mTORC2. Moreover, Torin1 and PP242 inhibited 4E-BP1 phosphorylation and cap-dependent mRNA translation, that both depend on mTORC1 activity, more efficiently than rapamycin [131,132].

As discussed previously, an important limitation of rapalogs is the removal of a negative feedback loop, resulting in the activation of PI3K/Akt signaling pathway which counteract the efficacy of rapalogs [87]. Akt requires the phosphorylation of the amino acid residues S473 and T308 to be fully activated. While mTORC2 phosphorylates Akt on S473, PDK-1 a downstream effector of PI3K regulates T308 phosphorylation. Specific mTOR kinase inhibitors have shown contradictory results on Akt phosphorylation. Whereas Ku-0063794 inhibited Akt S473 and T308 phosphorylation at the same concentration, AZD8055 required higher doses to block T308 phosphorylation [117,133]. In contrast, WYE-125132 inhibited S473 phosphorylation but had no effect on T308 [119]. This might be particularly relevant as T308 phosphorylated Akt is still able to activate a subset of downstream effectors even in the absence of S473 phosphorylation [134]. Therefore, additional studies are required to investigate the effects of specific mTOR kinase inhibitors on Akt activity. In contrast, dual PI3K/mTOR inhibitors consistently inhibited the phosphorylation of Akt on T308 and S473 and thus might have a stronger inhibitory effect on Akt than specific mTOR kinase inhibitors.

Finally, activation of Mek/Erk signaling pathway following exposure of cancer cells to ATP-competitive inhibitors of mTOR has also been reported even with dual PI3K/mTOR inhibitors [124]. This finding suggests that combining Mek and ATP-competitive inhibitors of mTOR may have additive anticancer effects. Consistent with this hypothesis, the anti-angiogenic efficacy of combined Mek and ATP-competitive inhibitors of mTOR is superior to either inhibitor alone [96]. A similar observation has been reported in a model of Waldenstrom macroglobulinemia [126].

In summary, these studies show that ATP-competitive inhibitors of mTOR display a stronger antiproliferative effect than rapalogs and also frequently induce cancer cell apoptosis, which is rarely observed with rapalogs. Current clinical studies are evaluating their efficacy in cancer patients [41].

## 8. Conclusions

Clinical trials have shown that the inhibition of mTORC1 by rapalogs has limited benefits in oncology when used in monotherapy. However, findings in basic research, that were confirmed in patients, have revealed that blocking mTOR induces the activation of proliferative and prosurvial signals that limit the anticancer efficacy of mTORC1 inhibitors. New therapeutic strategies that associate inhibitors of these proliferative and prosurvival signals in combination with mTORC1 inhibitors have proven their efficacies in experimental models and are being evaluated in ongoing clinical trials. In addition, the anticancer efficacy of ATP-competitive inhibitors of mTOR, that in contrast to rapalogs block both mTORC1 and mTORC2, are superior to rapalogs. Their toxicity and efficacy are being currently tested in clinical studies. Finally, emerging data show that the role of mTOR in cancer biology is not limited to the proliferating cancer cells but involves the tumor microenvironment. Particularly, the role of mTOR in innate and adaptive immune cells needs to be further explored in the context of cancer.
